# Selective and Irreversible Induction of Necroptotic Cell Death in Lung Tumorspheres by Short-Term Exposure to Verapamil in Combination with Sorafenib

**DOI:** 10.1155/2017/5987015

**Published:** 2017-10-19

**Authors:** Juan Sebastian Yakisich, Yogesh Kulkarni, Neelam Azad, Anand Krishnan V. Iyer

**Affiliations:** School of Pharmacy, Department of Pharmaceutical Sciences, Hampton University, Hampton, VA, USA

## Abstract

The presence of highly resistant cancer cells and the toxicity to normal cells are key factors that limit chemotherapy. Here, we used two models of highly resistant lung cancer cells: (1) adherent cells growing under prolonged periods of serum starvation (PPSS) and (2) cells growing as floating tumorspheres (FTs) to evaluate the effect of Verapamil (VP) in combination with Sorafenib (SF). Compared to cells growing under routine culture conditions (RCCs), PPPS cells or FTs were highly sensitive to short-term exposure (24 h) to VP 100 *μ*M + SF 5 *μ*M (VP100 + SF5). Recovery experiments exposing cells to VP100 + SF5 for 24 h followed by incubation in drug-free media for 48 h demonstrated that while PPSS as well as FT cells were unable to recover, cancer cells and the noncancerous cell line Beas-2B growing under RCCs were less sensitive and were also able to recover significantly. VP100 + SF5 induced significant changes in the expression of protein associated with apoptosis, autophagy, and to a lesser extent necroptosis. Coincubation experiments with z-VAD-FMK, necrostatin 1, or chloroquine showed evidence that necroptosis played a central role. Our data demonstrates that highly resistant cancer cells can be selectively eliminated by VP + SF and that necroptosis plays a central role.

## 1. Background

The toxicity of anticancer drugs to noncancer cells is an important barrier that limits the efficacy of anticancer drugs [1]. In addition, drug resistance of cancer cells due to mechanisms such as increased drug efflux, alteration or mutation of drug targets, alterations in DNA repair, and evasion of apoptosis [2] often limits the efficacy of anticancer drugs. The presence of a subpopulation of cancer stem cells (CSCs) or cancer stem-like cells (CS-LCs) associated with chemoresistance and tumor relapse has been also linked to poor response to chemotherapy in many cancers [3]. Novel therapeutic options that selectively target cancer cells, especially those with high resistance to anticancer drugs, with little or no toxicity to normal cells have been the focus of intensive research but the success has been limited. For instance, the success of targeted therapies that interfere with specific proteins involved in tumorigenesis rather than using broad base cancer treatments has been limited by the difficulty in identifying specific cancer biomarkers [4] and to the development of acquired drug resistance through mutations in targeted proteins or through the adaptation of alternate cancer cell survival strategies [5]. Drugs that more selectively target CSCs/CS-LCs have been identified but once again toxicity to normal cells limits the clinical application of these drugs. For instance, Salinomycin has been identified as a highly specific drug toward cancer stem cells [6] but its use in humans has been limited probably due to the considerable toxicity observed in mammals [7].

Tumorspheres are useful model for screening of drugs since they are enriched in cancer stem cells (CSCs) or cancer stem-like cells (CS-LCs) that are usually more resistant compared to non-CSCs/CS-LCs [8], and it is thought that the ability to form clonal spheres is a unique characteristic of CSCs [9, 10]. The ability to sustain proliferative signaling and divide in the absence of exogenous mitogenic stimulation leading to unregulated proliferation is considered one hallmark of cancer cells [11]. This has been demonstrated for glioma [12, 13], lung [14], and breast CSCs/CS-LCs [15] that can form spheres in serum-free media without exogenous mitogens. Lung tumorspheres (LTs) and mammospheres (MSs) obtained in the absence of any external mitogenic stimulation showed increased resistance to conventional anticancer drugs such as Paclitaxel (PX), hydroxyurea (HU), Colchicine (CX), and Obatoclax (OBT). We have also reported that adherent H460 lung and breast cancer cells that survive prolonged periods of serum starvation divide slowly and become highly resistant to PX, HU, CX, OBT, and the PI3 kinase inhibitors Wortmannin (WT) and LY294002 (LY) [16, 17]. LTs showed elevated expression of stemness-associated markers that may contribute to the multiresistant phenotype associated with CSCs/CS-LCs. On the other hand, the multiresistant phenotype of cells growing under PPSS is likely the result of extensive rewiring of signaling pathways rather than increased stemness [16]. These traits make cells growing under PPSS and tumorspheres useful complementary models to screen drugs able to overcome multidrug resistance as well as to identify the underlying mechanism(s). VP is a calcium channel blocker that has been shown to inhibit the activity of the MDR1 protein and has shown potential as a sensitizing agent to overcome the chemoresistance of CSCs/CS-LCs in a variety of cancers including lung [18], pancreatic [19], and breast [20] cancer cells. Sorafenib (SF) is a multikinase inhibitor that also inhibits the activity of the ABGC2 multidrug-resistant protein. However, combinatorial treatment using VP and SF has not been extensively characterized.

The aim of this study was to evaluate the effect of VP in combination with SF in lung cancer cells growing under PPSS as well as tumorspheres. We found that short term-exposure to VP + SF selectively and irreversibly decrease the viability, likely by activating necroptotic cell death, of cancer cells growing under PPSS or as tumorspheres but have little or negligible effect on noncancer cells or in cancer cells growing under RCCs.

## 2. Methods

### 2.1. Chemicals and Reagents

#### 2.1.1. Drugs

Verapamil (VP), z-VAD-FMK (zVAD), chloroquine (CQ), poly-HEMA (poly(2-hydroxyethyl methacrylate)), and MTT (thiazolyl blue tetrazolium bromide) were purchased from Sigma-Aldrich (St. Louis, MT). Sorafenib (SF) necrostatin 1 (Nec1), and 1-methyl-D-tryptophan (1-D-M-T) were purchased from VWR (Radnor, PA). Stock solutions of SF (10 mM), Nec1 (10 mM), and zVAD (10 mM) were in DMSO and stored in aliquots at −20°C. CQ was prepared as stock solution (10 mM) in distilled sterile water and filter sterilized and stored in aliquots at −20°C. VP (50 mM) was freshly prepared in distilled sterile water and filter sterilized. 1-D-M-T (20 mM stock solution) was prepared by dissolving in 0.1 N NaOH, and the pH was adjusted to 7.5 using hydrochloric acid, filter sterilized [21], and stored in aliquots at −20°C. Final dilutions were freshly prepared in culture media before use.

#### 2.1.2. Cell Culture

The human lung epithelial cancer cell line NCI-H460 and the noncancerous cell line Beas-2B were obtained from American Type Culture Collection (Manassas, VA). Beas-2B cells are epithelial cells that were isolated from normal human bronchial epithelium obtained from the autopsy of noncancerous individuals (http://www.atcc.org). For routine culture conditions (RCCs), cells were plated and propagated in complete media (CM) = RPMI 1640 (for NCI-H460) or DMEM/high glucose (for Beas-2B) supplemented with 5% FBS, L-glutamine, 100 U/ml penicillin, and 100 mg/ml streptomycin. Glutamine concentrations in RPMI-1640 and DMEM/high-glucose media were 2 or 4 mM, respectively. All cells were cultured in a 5% CO_2_ environment at 37°C. For cells growing under routine culture conditions (PPSS) or growing as floating tumorspheres, cells were maintained in serum-free media (same as CM but without FBS, see details below).

#### 2.1.3. Generation of Lung Tumorspheres (LTs)

A detailed protocol for the generation of floating tumorspheres grown in the absence of any external mitogenic stimulation can be found in Yakisich et al. [15]. Briefly, H460 cells grown in CM (70–80% confluency) were cultured overnight in serum-free media (SFM, same as CM but without FBS). Then, cells were trypsinized and incubated in SFM for at least 14 days in poly-HEMA-coated plated to prevent attachment. For maintenance of LTs, the SFM was replaced every 3-4 days. LTs grown in SFM for 14–21 days were used for subsequent experiments.

#### 2.1.4. Short-Term Antiproliferative Assay (MTT Assay and CCK Assay)

For routine culture conditions and adherent cultures (parental H460 and Beas-2B), cells were plated in 96-well cell-culture microplates (Costar, USA) at ~2000 cells per well and incubated overnight in CM. For cells growing under prolonged periods of serum starvation (PPSS), cells (~500 cells/well) were plated in 96-well cell-culture microplates and incubated overnight in CM to allow them to adhere and then maintained in SFM for 7–12 days. Then, the cells were exposed to the appropriate concentration of drug or vehicle for 24–72 h. Cell viability for adherent cells was evaluated by the MTT assay. The absorbance of solubilized formazan was read at 570 nm using Gen 5 2.0 All-In-One microplate reader (Bio-TEK, Instruments Inc.). For floating LTs and MSs, cells growing in poly-HEMA plates were collected in 15 ml Falcon tubes, centrifuged at 700 rpm × 3 min, and resuspended in fresh SFM. In order to plate the same number of cells, this cell suspension was split in 1 ml aliquots. Vehicle or drugs were added to each aliquot and then 150 *μ*l cell suspension was loaded into each microwell (in a 96-well plate) and incubated for 72 h. For floating LTs, cell viability was evaluated by the CCK-8 assay (Dojindo Laboratories).

In all cases, the highest concentration of DMSO was used in the control and this concentration was maintained below 0.01% (*v*/*v*). This DMSO concentration did not show any significant antiproliferative effect on the cell lines or tumorspheres in a short-term assay.

#### 2.1.5. Western Blotting

Preparation of cell lysates and Western blotting were performed as described previously [22]. Antibodies for PARP, cleaved PARP, caspase 3, caspase 9, RIP1, MLKL, Beclin, p62, and peroxidase-conjugated secondary antibody were purchased from Cell Signaling (Danvers, MA). Antibody for GAPDH was purchased from Santa Cruz Biotechnology (Dallas, TX). The blotting membranes were probed with 1 : 1000 diluted primary antibody and 1 : 4000 for the peroxidase-conjugated secondary antibody. Immune complexes were detected by chemiluminescence using SuperSignal™ West Femto Maximum Sensitivity Substrate (Thermo Fisher Scientific, Grand Island, NY) and photographed using myECL imager instrument (Thermo Fisher Scientific, Grand Island, NY).

#### 2.1.6. Statistical Analysis

All pairwise multiple comparison procedures (ANOVA, Student-Newman-Keuls method) have been done using SigmaPlot (V. 11.0) software.

## 3. Results

### 3.1. Short-Term Exposure to Verapamil in Combination with Sorafenib Inhibits the Viability of Highly Resistant Cancer Cell

**Figure 1:**
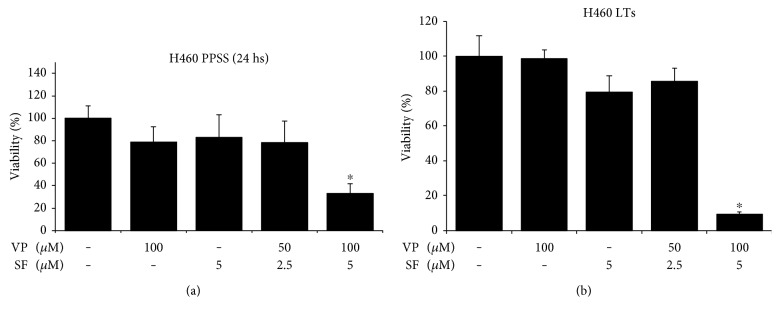
VP in combination with SF significantly decreases the viability of cells growing under PPSS or as FTs. (a) Cells growing under PPSS for 8–10 days were incubated with the indicated concentrations of VP and SF alone or in combination for 24 h. Cell viability was measured by the MTT assay. (b) Cells growing as FTs for 14–16 days were incubated with the indicated concentrations of VP and SF alone or in combination for 24 h. Cell viability was measured by the CCK assay. Results (*X* ± SD) are representative of two independent experiments performed in sextuplicate. ∗ indicates *P* < 0.01 (ANOVA).

We first investigated the ability of VP + SF to inhibit the viability of human lung H460 cancer cells growing under culture conditions that promote stemness and make cells highly resistant to anticancer agents: (1) cells growing under prolonged periods of serum starvation (PPSS) and (2) cells growing as floating tumorspheres (FTs). Cells growing under PPSS for 8 days were incubated for 24 hours with VP (100 *μ*M), SF (5 *μ*M), VP (50 *μ*M) + SF (2.5 *μ*M), or VP (100 *μ*M) + SF (5 *μ*M), and viability was measured by the MTT assay.  Figure 1(a) shows that VP or SF alone or low concentration of VP (50 *μ*M) + SF (2.5 *μ*M) has no significant effect on cell viability but high concentration of VP (100 *μ*M) + SF (5 *μ*M) significantly decrease the viability of H460 cancer cells. A similar effect was observed when these drugs were tested in H460 LTs, and viability was measured by the CCK assay (Figure 1(b)). As it can be observed in Figure S1 available online at https://doi.org/10.1155/2017/5987015, in LTs treated for 24 h with DMSO alone in 96-well uncoated microplates, the cells are able to reattach. In contrast, VP (100 *μ*M) + SF (5 *μ*M) treated cells fail to reattach and lose integrity (Figure S1) indicating that this drug combination induces a rapid cell death that is in agreement with the massive decrease in cell viability measured by the MTT and CCK assays in PPSS and FTs, respectively.

### 3.2. Short-Term Exposure to Verapamil in Combination with Sorafenib Has Little Effect on the Viability of Cancer and Noncancer Cells Growing under Routine Culture Conditions

**Figure 2:**
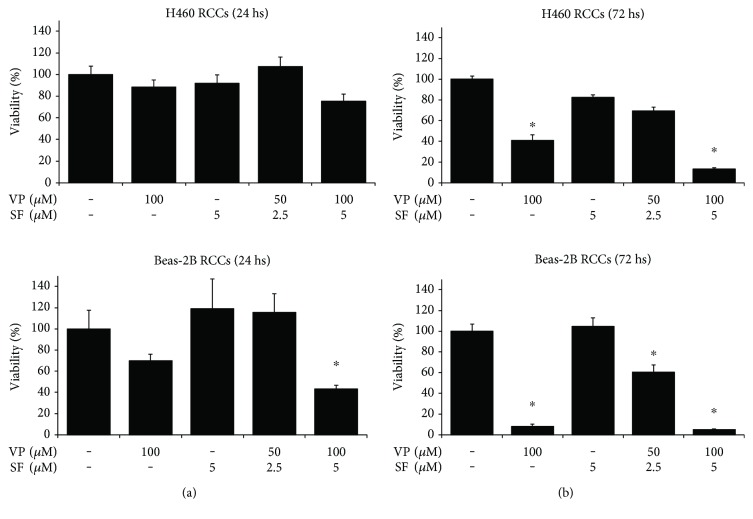
Cancer cells (H460) and the noncancerous cell line Beas-2B growing under RCCs are relatively resistant to short-term exposure to VP in combination with SF. Cells growing under RCCs were incubated with the indicated concentrations of VP and SF alone or in combination for 24 h (a) or 72 h (b). Cell viability was measured by the MTT assay. Results (*X* ± SD) are representative of two independent experiments performed in sextuplicate. ∗ indicates *P* < 0.01 (ANOVA).

In order to evaluate the effect of VP, SF, and VP + SF on cancer cells (H460) and noncancer cells (Beas-2B) growing under RCCs, a culture condition in which cancer cells are relatively highly sensitive to anticancer drugs and have low expression levels of stemness-associated markers, cells growing under RCCs were incubated for 24 hours with VP (100 *μ*M), SF (5 *μ*M), VP (50 *μ*M) + SF (2.5 *μ*M), or VP (100 *μ*M) + SF (5 *μ*M), and viability was measured by the MTT assay.  Figure 2(a) shows that both cancer and noncancer cells growing under RCCs are more resistant to VP + SF compared to cells growing under PPSS or as FTs (see Figure 1). In parallel, we tested the effect of 72 hours of exposure to VP or SF alone or in combination (Figure 2(b)).

### 3.3. Short-Term Exposure to Verapamil in Combination with Sorafenib Irreversibly Inhibits the Viability of Lung Cancer Cells Growing under Prolonged Periods of Serum Starvation (PPSS)

**Figure 3:**
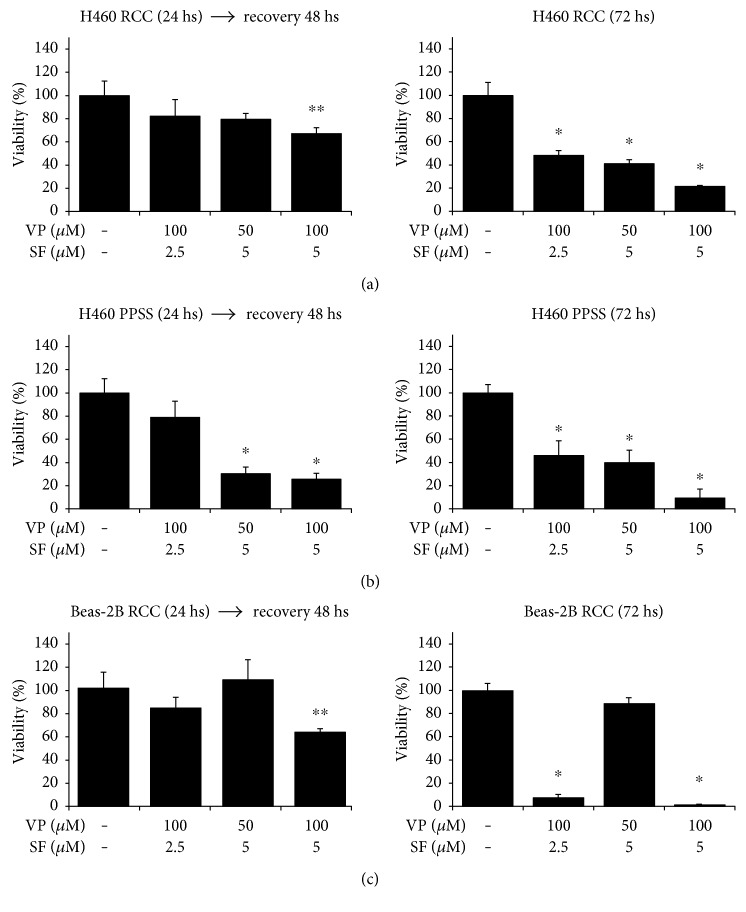
Short-term exposure to VP + SF irreversibly and selectively decreases the viability of cancer cells (H460) growing under RCCS as well as under PPSS. (a) H460 cells growing under RCCs were incubated with the indicated concentration of drugs for 24 h and then allowed to recover in drug-free media for 48 h (left panel). In parallel, cells were treated with the same drugs for 72 h (right panel). Cell viability was measured by the MTT assay. (b) H460 cells growing under PPSS (8–10 days) were incubated with the indicated concentration of drugs for 24 h and then allowed to recover in drug-free media for 48 h (left panel). In parallel, cells were treated with the same drugs for 72 h (right panel). Cell viability was measured by the MTT assay. (c) Beas-2B cells growing under RCCs were incubated with the indicated concentration of drugs for 24 h and then allowed to recover in drug-free media for 48 h (left panel). In parallel, cells were treated with the same drugs for 72 h (right panel). Results (*X* ± SD) are representative of two independent experiments performed in sextuplicate. A simplified schema for the recovery experiments (left panels in a, b, and c) and continuous treatment with drugs for 72 h (right panels in a, b, and c) is shown in Figure S2). ∗ and ∗∗ indicate *P* < 0.01 and *P* < 0.05, respectively (ANOVA).

To evaluate if the effect of short-term exposure to VP + SF can irreversibly decrease the viability of cancer cells, we performed “recovery” experiments and compared to “continuous treatment” experiments as indicated in Figure S2. For “recovery” experiments, cells growing under RCCs and cells growing under PPSS for 8 days were treated with different concentrations of VP + SF (VP 100 *μ*M + SF 2.5 *μ*M; VP 50 *μ*M + SF 5 *μ*M; or VP 100 *μ*M + SF 5 *μ*M). After 24 h treatment, the drug was removed, cells were allowed to recover in drug-free media for 48 h, and viability was evaluated by the MTT assay.  Figure 3 (left panels) shows that cells treated for 24 h and then allowed to recover for 48 h in drug-free media cannot recover showing decreased viability compared to control cells. In parallel experiments (“continuous treatment” experiments), cells were treated with VP + SF for 72 hs (Figure 3, right panels). Overall, these results indicate that short exposure (24 h) to VP + SF is able to irreversibly induce cell death in cancer cells growing under PPSS but both cancer cells and noncancer cells (Beas-2B) can recover significantly from short-term exposure to VP + SF. In addition, we performed the same “recovery” experiments but allowing cells to recover for extended periods (up to 5 days for cells treated with VP100 + SF5 for 24 h) in drug-free media to ensure that cells growing under RCCs continue to recover and are not irreversibly damaged as PPSS cells. In these set of experiments, the MTT assay will not be reliable due to the high number of cells expected in control wells after ~6 days of culture. Instead, at the end of the experiments, cells were stained with Hema 3® Stain Set according to manufacturer's instructions (Fisher Diagnostics, Middletown, VA) and evaluated microscopically.  Figure 3(a) shows that when cells growing under RCCs were treated for only 24 h with VP100 + SF5 and then allowed to recover for 5 days they were able to grow continuously although at lower density compared to control cells. Longer treatment (for 72 h) completely eliminated both Beas-2B and H460 cells. In contrast, in cells growing under PPSS for 9 days both, short (24 h) and long (72 h) treatments, completely eliminated H460 cells (Figure 3(b)). These results are consistent with the MTT data shown in Figure 3.

### 3.4. VP + SF Modulates the Expression of Key Proteins Involved in Apoptosis, Autophagy, and Necroptosis in a Cell Type-Dependent Manner

**Figure 4:**
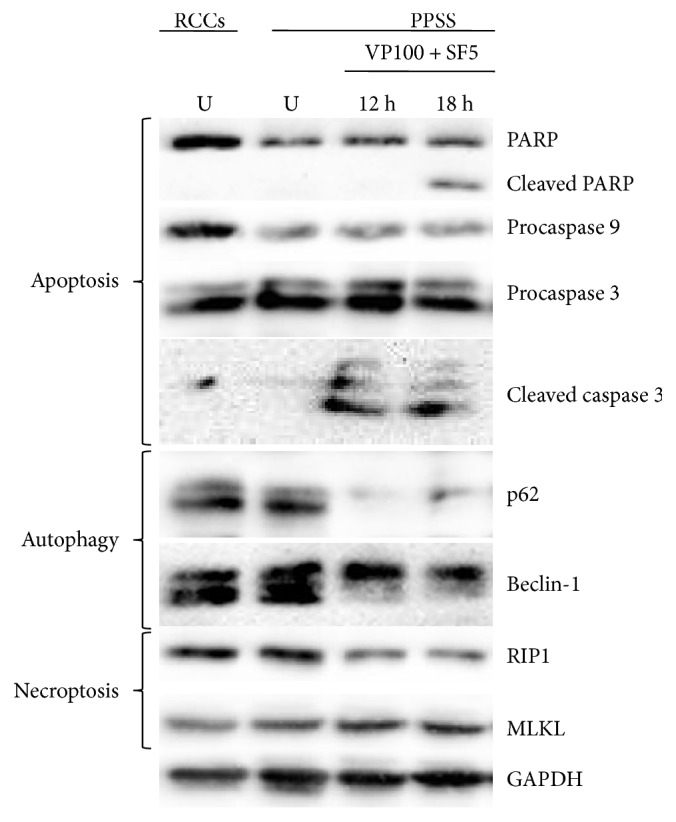
VP + SF modulates the expression of key protein involved in apoptosis (PARP, cleaved PARP, procaspase 9, procaspase 3, and cleaved caspase 3), autophagy (p62 and Beclin), and necroptosis (RIP1 and MLKL). Cells were grown under routine culture conditions (RCC) or under prolonged periods of serum starvation (PPSS, for 8 days). PPSS cells were treated with VP (100 *μ*M) + SF (5 *μ*M) for 12 or 18 hs. Control (untreated) cells (U) were incubated with equivalent concentrations of DMSO.

To gain insight into the mechanism by which VP + SF eliminates cancer cells, we evaluated the expression of key proteins involved in apoptosis (PARP, caspase 3, and caspase 9), autophagy (Beclin-1 and p62), and necroptosis (RIP1 and MLKL). Protein lysates were collected from floating and attached H460 cells grown under PPSS for 8 days that were exposed for 12 or 18 hs to VP 100 *μ*M + SF 5 *μ*M, and the expression of proteins was evaluated by Western blots. Control cells were treated with equivalent concentrations of vehicle (H_2_O + DMSO for 18 hs). For comparison, the expression of these protein markers was also evaluated in untreated cells growing under RCCs.  Figure 4 shows that VP100 + SF5 modulates the expression of these key proteins in cells growing under PPSS. For instance, the apoptosis markers (cleaved caspase 3 and cleaved PARP) were found to be elevated. VP100 + SF5 significantly decreased the expression of the autophagy markers p62 and Beclin-1. The necroptosis markers' RIP1 levels were reduced significantly. Collection of protein lysates for Western blots was not performed at later times (e.g., 24 h) since microscopic observation showed extensive cell death (in agreement with the viability data presented in Figure 1) and loss of cellular integrity.

### 3.5. Necrostatin 1 (Nec1) Partially Prevented the Effects of VP + SF on Cell Viability

**Figure 5:**
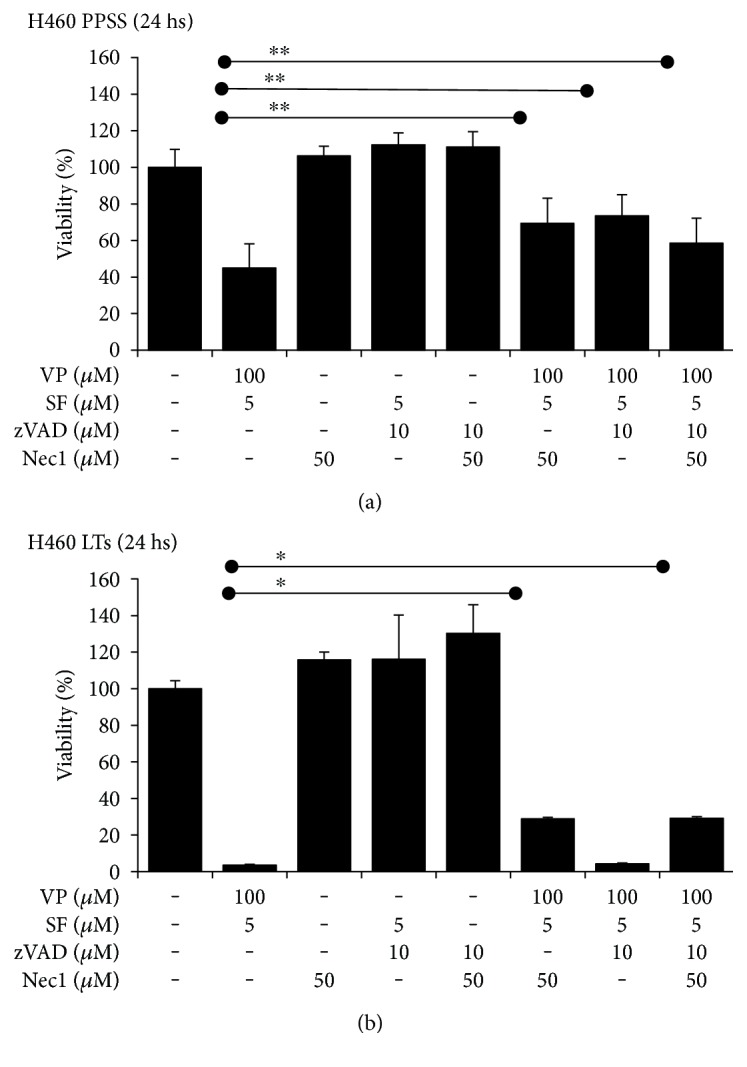
zVAD and Nec1 partially rescued the viability of cells. (a) Cells growing under PPSS for 8–10 days were incubated with the indicated concentrations of VP and SF alone or in the presence of zVAD or Nec1 for 24 h. Cell viability was measured by the MTT assay. (b) Cells growing as FTS for 14–16 days were incubated with the indicated concentrations of VP and SF alone or in the presence of zVAD or Nec1 for 24 h. Cell viability was measured by the CCK assay. Results (*X* ± SD) are representative of two independent experiments performed in sextuplicate. ∗ and ∗∗ indicate *P* < 0.01 and *P* < 0.05, respectively (ANOVA).

**Figure 6:**
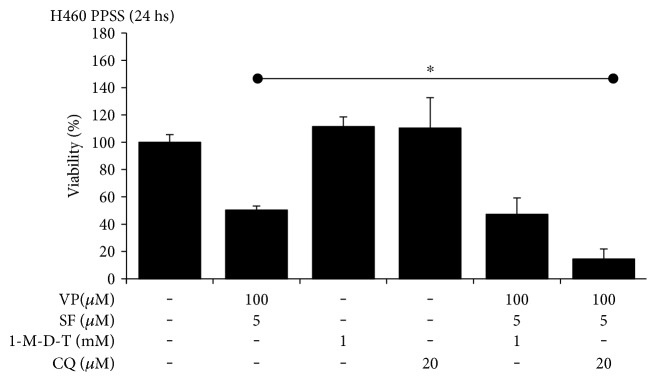
Chloroquine potentiates VP + SF effects on cell viability. Cells growing under PPSS for 8–10 days were incubated with VP (100 *μ*M) + SF (5 *μ*M) alone or in the presence of 1-M-D-T or CQ for 24 h. Cell viability was measured by the MTT assay. Results (*X* ± SD) are representative of two independent experiments performed in sextuplicate. ∗ indicates *P* < 0.01 (ANOVA).

Due to the difficulty in monitoring the expression profiles of proteins at later times, we used pharmacological inhibitors of apoptosis (zVAD-FMK (zVAD)), necroptosis (necrostatin 1 (Nec1)), or autophagy (chloroquine (CQ)) to further elucidate the mechanism of cell death triggered by VP + SF. H460 cancer cells growing under PPSS for 8–10 days were incubated with VP 100 *μ*M + SF 5 *μ*M alone or coincubated with Nec1 (50 *μ*M) and zVAD (10 *μ*M). Nec1 and zVAD partially rescued the viability cells when incubated for 24 hours with VP 100 *μ*M + SF 5 *μ*M. This protective effect was observed in cells growing under PPSS (Figure 5(a)). In cells growing as FTs, Nec1 but not ZVAD partially prevented the effect of VP + SF on cell viability (Figure 5(b)). The autophagy inhibitor CQ had no protective effect on the decrease of cell viability induced by VP + SF in either cell growing under PPSS (Figure 6) or as FTs (Figure S4). In fact, in cells growing under PPSS, CQ significantly enhanced the effect of VP 100 *μ*M + SF 5 *μ*M (Figure 6). Since Nec1 is also an IDO inhibitor, the effect of 1-methyl-D-tryptophan (1 mM), a classical IDO inhibitor that does not affect necroptosis [23], was tested and showed no protective effect in cells growing under PPSS (Figure 6) or as FTs (data not shown).

## 4. Discussion

Lung cancer is a leading cause of cancer-related deaths [24, 25], and resistance to chemotherapy is a major challenge to treat these tumors. Therefore, a drug or treatment that can selectively kill cancer cells with no harm to normal cells has been considered the magic bullet to treat these malignancies. In this study, we evaluated the anticancer effects of Verapamil in combination with Sorafenib (VP + SF) in lung cancer cells growing under three different culture conditions: routine culture conditions (RCCs), prolonged periods of serum starvation (PPSS), and cell growing as floating tumorspheres (FTs). FTs growing in absence of external mitogenic factors showed elevated resistance to conventional anticancer drugs such as PX, CX, and HU [14] which is a trait usually found in CSCs/CS-LCs [9]. Lung CSCs are known to be resistant to PX [26] and other conventional anticancer drugs such as Cisplatin, Doxorubicin, and Etoposide [27]. In the present study, we found that both VP and SF, even at high concentrations (100 *μ*M and 5 *μ*M, resp.) were almost ineffective when used as a single drug. When used in combination, only a high concentration of VP + SF (100 *μ*M + 10 *μ*M, resp.) showed a potent inhibitory effect on cell viability in cells growing under PPSS (Figure 1(a)) as well as in cells growing as floating tumorspheres (Figure 1(b)). This effect was observed within 24 hours of exposure. More importantly, (1) cancer cells and noncancer cells growing under RCCs were much more resistant to 24 hours exposure to VP (100 *μ*M) + SF (5 *μ*M) and (2) the effect of 24 h exposure to VP (100 *μ*M) + SF (5 *μ*M) on cell viability was found to be irreversible in cancer cells growing under PPSS. In contrast, in cancer cells and noncancer cells growing under RCCs, the effect was reversible since cells were able to recover once the VP + SF was removed from the media (Figure 3). This indicates that VP (100 *μ*M) + SF (5 *μ*M) triggers cell death only in cells growing under PPSS in an irreversible manner within 24 h. For technical reasons, the reversibility was tested only in adherent cells (RCCs and PPSS) where media can be easily replaced without the need of centrifugation steps that would be required for FTs.

In our study, we used a very high concentration of VP: 50–100 *μ*M. The average steady-state plasma levels measured for Verapamil was approximately 0.5 *μ*M [28]. However, Verapamil has been used *in vitro* as a classical inhibitor of MDR1 at 50 *μ*M [18, 29] and up to 200 *μ*M [19]. In humans, SF achieves drug levels of about 10 *μ*M [30] and this concentration is enough to inhibit ABCG2 [31]. It is important to mention that the mechanism by which elevated concentrations of VP + SF when used in combination decrease the viability of highly resistant cancer cells may be unrelated to their ability to downregulate the expression of MDR1 or ABCG2, respectively. The explanation behind this assumption is because we previously reported that cancer cells growing under PPSS respond aberrantly and sometimes paradoxically to a variety of pharmacological agents. For instance, in H460 cells growing under PPSS, we found that VP and SF (alone or in combination) induce rather than decrease the expression of MDR1 and ABCG2, respectively. Similarly, Obatoclax (a Bcl-2 inhibitor) downregulated the expression Bcl-2 in cells growing under RCCs but induced a paradoxical increase in the levels of this protein in cells growing under PPSS [16]. We attributed this response to a “rewiring” of signaling pathways as an adaptative response of cells to prolonged serum starvation, and it is likely that similar changes may occur in FTs that typically grow in serum-free conditions. Additional experiments behind the scope of this manuscript are needed to better understand the biology of these models of chemoresistance in order to elucidate the mechanism by which VP + SF exerts the potent effects reported in the present study. This new knowledge will be valuable for the identification or development of new toxic compounds with similar activities against highly resistant cancer cells. Regardless of the high concentration of VP used in our study that may limit its clinical use, our data provide significant novel insight into the biology of multiresistant cancer cells. First, we have identified a combination of drugs that does not need prolonged exposure to selectively and irreversibly eliminate chemoresistant cells (such as CSCs/CS-LCs) while having little or no effects in non-CSCs/CS-LCs as well as in noncancerous cells (e.g., Beas-2B). This finding can be exploited to develop chemotherapy regimens to irreversibly induce cell death in chemoresistant cancer cells by short-term treatment (24 h). Second, at the molecular level, we showed that VP + SF modulated the expression of key proteins involved in apoptosis, autophagy, and necroptosis (Figure 4) but no conclusive data could be obtained regarding the type of cell death triggered by this compound combination by analyzing the protein profile. While VP + SF increased the expression of cleaved PARP and cleaved caspase 3 in H460 cells, this treatment reduced the levels of P62 and Beclin-1 and RIP1 suggesting that autophagy may play a protective role. However, pharmacological evidence indicates that necroptosis and, to a lesser extent, apoptosis play a major role in cell death induced by VP + SF since (1) the inhibitory effect of VP + SF on cell viability could be partially prevented by Nec1 in both cells growing under PPSS and tumorspheres. zVAD, a pan caspase inhibitor, partially prevented the effect of VP + SF in cells growing under PPSS but had no effect in FTs (Figures 5(a) and 5(b)), (2) the autophagy inhibitor CQ did not prevented the effect of VP + SF neither in cells growing under PPSS nor in cells growing as FTS. Moreover, the significant enhancement on the decrease of cell viability when CQ was added to VP + SF (Figure 6) strongly suggests that induction of autophagy plays a protective role in VP + SF-induced cell death. Finally, the lack of effect of the IDO inhibitor 1-M-D-T indicates that Nec1 exerts its protective effect likely via inhibition of RIP1. Overall, our results reveal a complex crosstalk between apoptosis, necroptosis, and autophagy and support a model in which necroptosis has an important role in the cell death triggered by VP + SF. The differential protective effect of zVAD in cells growing under PPSS compared to FTs needs further evaluation. One possible explanation is that in our experimental system for cells growing under PPSS and cells growing as FTS, we use only serum-free media without any external mitogenic stimulation (e.g., EGF or bFGF) and the length of the lack of mitogenic factors rewires the cell death machinery to a different status depending on how long the cells have been adapted to SFM conditions. This hypothesis is supported by the differential protein levels of PARP and procaspase 9 observed in cells growing under PPSS compared with cells growing under RCCs (Figure 4). We have previously demonstrated that cells growing under PPSS rewire signaling pathways associated with multidrug resistance and respond aberrantly to inhibitors of multidrug resistance proteins such as MDR1 [16].

Our results clearly demonstrate that VP + SF by their ability to eliminate highly resistant cancer cells can be a leading combination to elucidate the underlying mechanism(s) that is necessary to selectively eliminate highly resistant cancer cells responsible for chemoresistance and tumor relapse.

## 5. Conclusion

We report for the first time that cancer cells growing under PPSS or growing as FTs display a multidrug-resistant phenotype are, compared to cells growing under culture conditions, highly sensitive to a combination of VP + SF. We presented pharmacological evidence that short exposure to VP + SF irreversibly triggers necro/apoptotic cell death in cells growing under PPSS and necroptotic cell death in cells growing as FTs. More importantly, noncancer cells can almost fully recover from short-term exposure to VP + SF. Therefore, we have identified a novel therapeutic opportunity that can be considered an “Achilles' heel” and can be targeted to selectively kill highly resistant cancer cells including CSCs/CS-LCs responsible for tumor resistance and tumor relapse while sparing noncancer cells.

## Supplementary Material

Figure S1. Representative images of LTs treated for 24 h with DMSO alone (control) or VP (100 µM) + SF (5 µM). Magnification: 20X (Bar = 200 µM). The inset shows an example of cell that reattach to the plate when treated with DMSO alone. In contrast, in the drug-treated plate, cells fail to reattach and clearly shows loss of cellular integrity. Figure S2. Simplified schema for the “Recovery” (A) and “Continuous” treatment (B) experiments performed for figure 3. For recovery experiments control or experimental cells (Exp.) were treated with DMSO or Verapamil+Sorafenib (VP+SF), respectively. After 24 h the media was changed (MC), incubated with drug-free media (Media lone) for 48 h and cell viability was measured at 72 h. For “Continuous” treatment” control or experimental cells (Exp.) were treated with DMSO or Verapamil+Sorafenib (VP+SF), respectively and cell viability was measured at 72 h. Figure S3a. Representative images of Beas-2B and H460 cells growing under RCCs and then treated for 72 h with DMSO alone (control, top pictures) or VP (100 µM) + SF (5 µM) (VP100+SF5) for 24 h (middle pictures) or 72 h (bottom pictures) followed by incubation in drug-free media for up to 5 days (for cells treated for 24h). Magnification: 20X. The results clearly shows that Beas-2B and H460 cells treated for 24 h with VP100+SF5 are able to recover while treatment for 72 h is toxic leaving only cellular debris. Figure S3b. Representative images of H460 cells growing under PPSS for 9 days and then treated for 72 h with DMSO alone (control, top picture, left) or VP (100 µM) + SF (5 µM) (VP100+SF5) for 24 h (middle picture, left) or 72 h (bottom picture, left) followed by incubation in drug-free media for up to 5 days (for cells treated for 24h). Magnification: 20X. The insets show examples of control cells (top picture, right) and cell debris (bottom picture, right) showing that treatment with VP100+SF5 for 24 h or 72 h irreversible eliminates H460 cells growing under PPSS. Figure S4. *CQ does not prevent VP+SF decrease on cell viability in FTs*. Cells growing as FTS for 14-16 days were incubated with VP (100 µM) + SF (5 µM) alone or in the presence of CQ for 24 h. Cell viability was measured by the CCK assay. Results (X±SD) are representative of two independent experiments performed in sextuplicates.

## Abbreviations

RCCs: Routine culture conditions
PPSS: Prolonged period of serum starvation
FTs: Floating tumorspheres
VP: Verapamil
SF: Sorafenib
CQ: Chloroquine
zVAD: z-VAD-FMK
Nec1: Necrostatin 1
1-M-D-T: 1-Methyl-D-tryptophan.
