# Supplementary material for: Selective and Irreversible Induction of Necroptotic Cell Death in Lung Tumorspheres by Short-Term Exposure to Verapamil in Combination with Sorafenib
Source: Stem Cells Int. 2017 Oct 19;2017:5987015. doi: 10.1155/2017/5987015 (PMC5671752; doi:10.1155/2017/5987015)
Supplement: Supplementary file 1 — Figure S1. Representative images of LTs treated for 24 h with DMSO alone (control) or VP (100 µM) + SF (5 µM). Magnification: 20X (Bar = 200 µM). The inset shows an example of cell that reattach to the plate when treated with DMSO alone. In contrast, in the drug-treated plate, cells fail to reattach and clearly shows loss of cellular integrity. Figure S2. Simplified schema for the “Recovery” (A) and “Continuous” treatment (B) experiments performed for figure 3. For recovery experiments control or experimental cells (Exp.) were treated with DMSO or Verapamil+Sorafenib (VP+SF), respectively. After 24 h the media was changed (MC), incubated with drug-free media (Media lone) for 48 h and cell viability was measured at 72 h. For “Continuous” treatment” control or experimental cells (Exp.) were treated with DMSO or Verapamil+Sorafenib (VP+SF), respectively and cell viability was measured at 72 h. Figure S3a. Representative images of Beas-2B and H460 cells growing under RCCs and then treated for 72 h with DMSO alone (control, top pictures) or VP (100 µM) + SF (5 µM) (VP100+SF5) for 24 h (middle pictures) or 72 h (bottom pictures) followed by incubation in drug-free media for up to 5 days (for cells treated for 24h). Magnification: 20X. The results clearly shows that Beas-2B and H460 cells treated for 24 h with VP100+SF5 are able to recover while treatment for 72 h is toxic leaving only cellular debris. Figure S3b. Representative images of H460 cells growing under PPSS for 9 days and then treated for 72 h with DMSO alone (control, top picture, left) or VP (100 µM) + SF (5 µM) (VP100+SF5) for 24 h (middle picture, left) or 72 h (bottom picture, left) followed by incubation in drug-free media for up to 5 days (for cells treated for 24h). Magnification: 20X. The insets show examples of control cells (top picture, right) and cell debris (bottom picture, right) showing that treatment with VP100+SF5 for 24 h or 72 h irreversible eliminates H460 cells growing under PPSS. Figure S4 [file 5987015.f1.pdf]

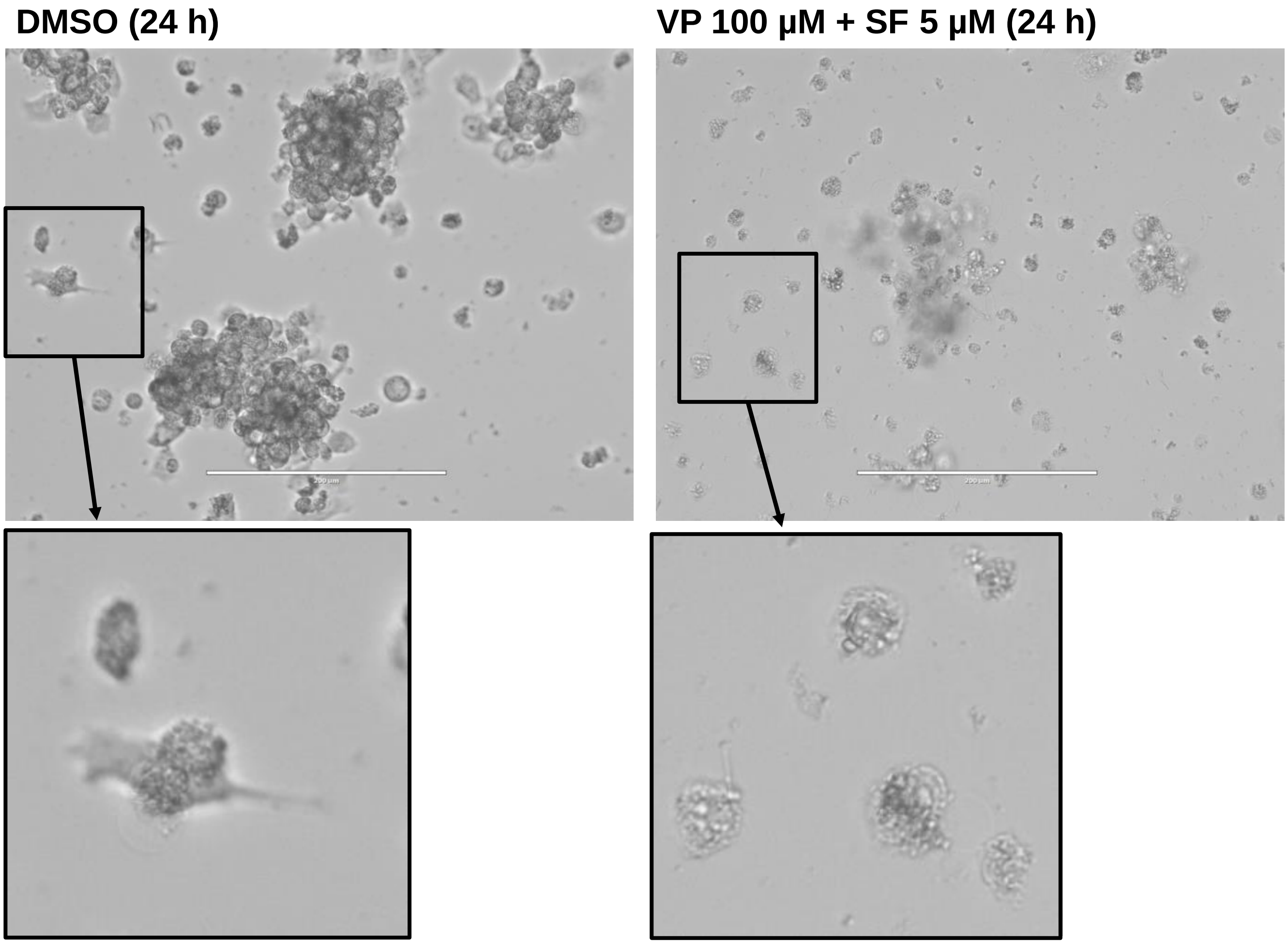

Figure S1. Representative images of LTs treated for 24 h with DMSO alone (control) or VP (100 µM) + SF (5 µM). Magnification: 20X (Bar = 200 µM). The inset shows an example of cell that reattach to the plate when treated with DMSO alone. In contrast, in the drug-treated plate, cells fail to reattach and clearly shows loss of cellular integrity.

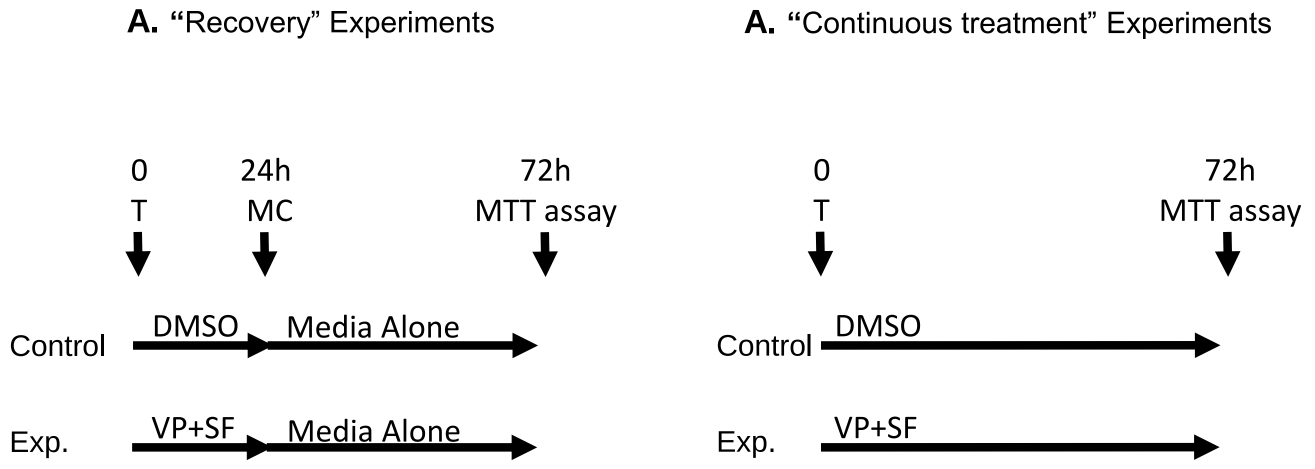

Figure S2. Simplified schema for the "Recovery" (A) and "Continuous" treatment (B) experiments performed for figure 3. For recovery experiments control or experimental cells (Exp.) were treated with DMSO or Verapamil+Sorafenib (VP+SF), respectively. After 24 h the media was changed (MC), incubated with drug-free media (Media lone) for 48 h and cell viability was measured at 72 h. For "Continuous" treatment" control or experimental cells (Exp.) were treated with DMSO or Verapamil+Sorafenib (VP+SF), respectively and cell viability was measured at 72 h.

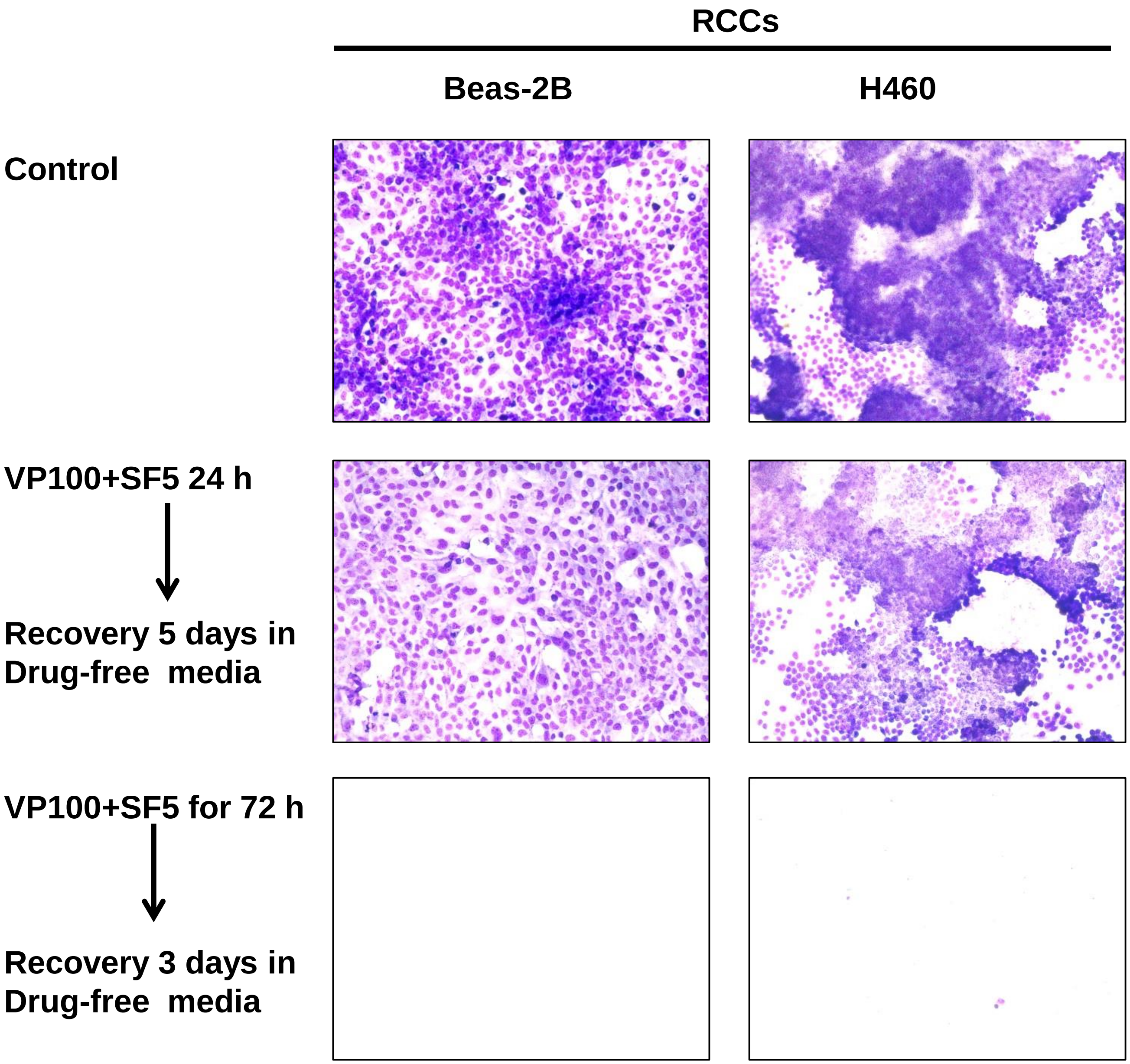

Figure S3a. Representative images of Beas-2B and H460 cells growing under RCCs and then treated for 72 h with DMSO alone (control, top pictures) or VP (100 µM) + SF (5 µM) (VP100+SF5) for 24 h (middle pictures) or 72 h (bottom pictures) followed by incubation in drug-free media for up to 5 days (for cells treated for 24h). Magnification: 20X. The results clearly shows that Beas-2B and H460 cells treated for 24 h with VP100+SF5 are able to recover while treatment for 72 h is toxic leaving only cellular debris.

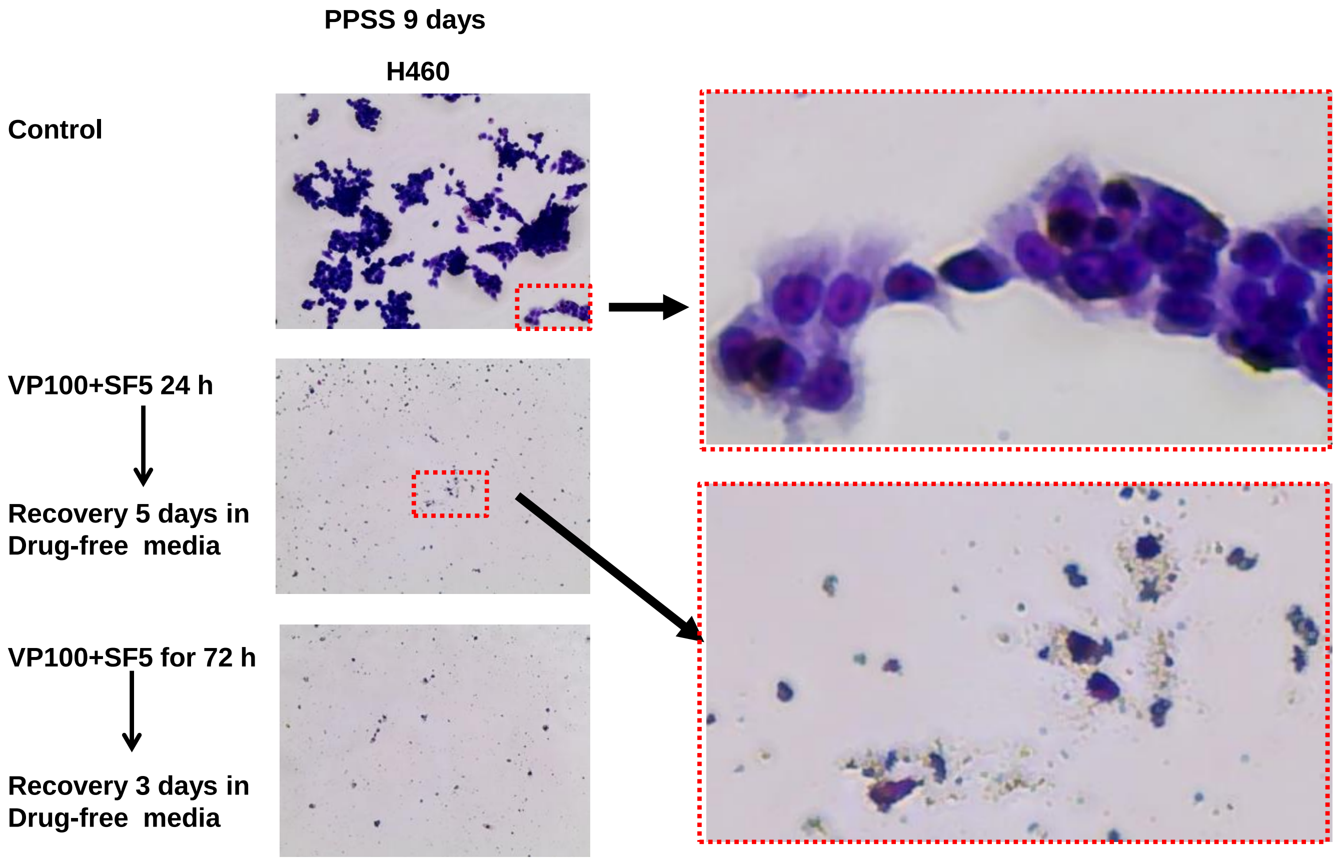

Figure S3b. Representative images of H460 cells growing under PPSS for 9 days and then treated for 72 h with DMSO alone (control, top picture, left) or VP (100 µM) + SF (5 µM) (VP100+SF5) for 24 h (middle picture, left) or 72 h (bottom picture, left) followed by incubation in drug-free media for up to 5 days (for cells treated for 24h). Magnification: 20X. The insets show examples of control cells (top picture, right) and cell debris (bottom picture, right) showing that treatment with VP100+SF5 for 24 h or 72 h irreversible eliminates H460 cells growing under PPSS.

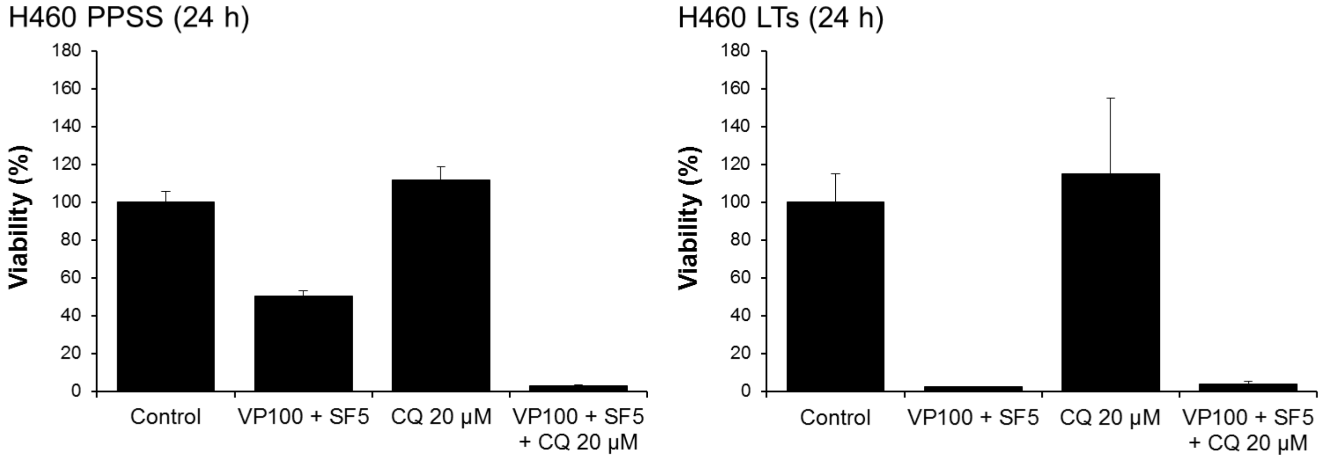

Figure S4. *CQ does not prevent VP+SF decrease on cell viability in FTs*. Cells growing as FTS for 14-16 days were incubated with VP (100 µM) + SF (5 µM) alone or in the presence of CQ for 24 h. Cell viability was measured by the CCK assay. Results (X±SD) are representative of two independent experiments performed in sextuplicates.
