# Supplementary figures and images for: Selective and Irreversible Induction of Necroptotic Cell Death in Lung Tumorspheres by Short-Term Exposure to Verapamil in Combination with Sorafenib
Source: Stem Cells Int. 2017 Oct 19;2017:5987015. doi: 10.1155/2017/5987015 (PMC5671752; doi:10.1155/2017/5987015)

## Slide 1
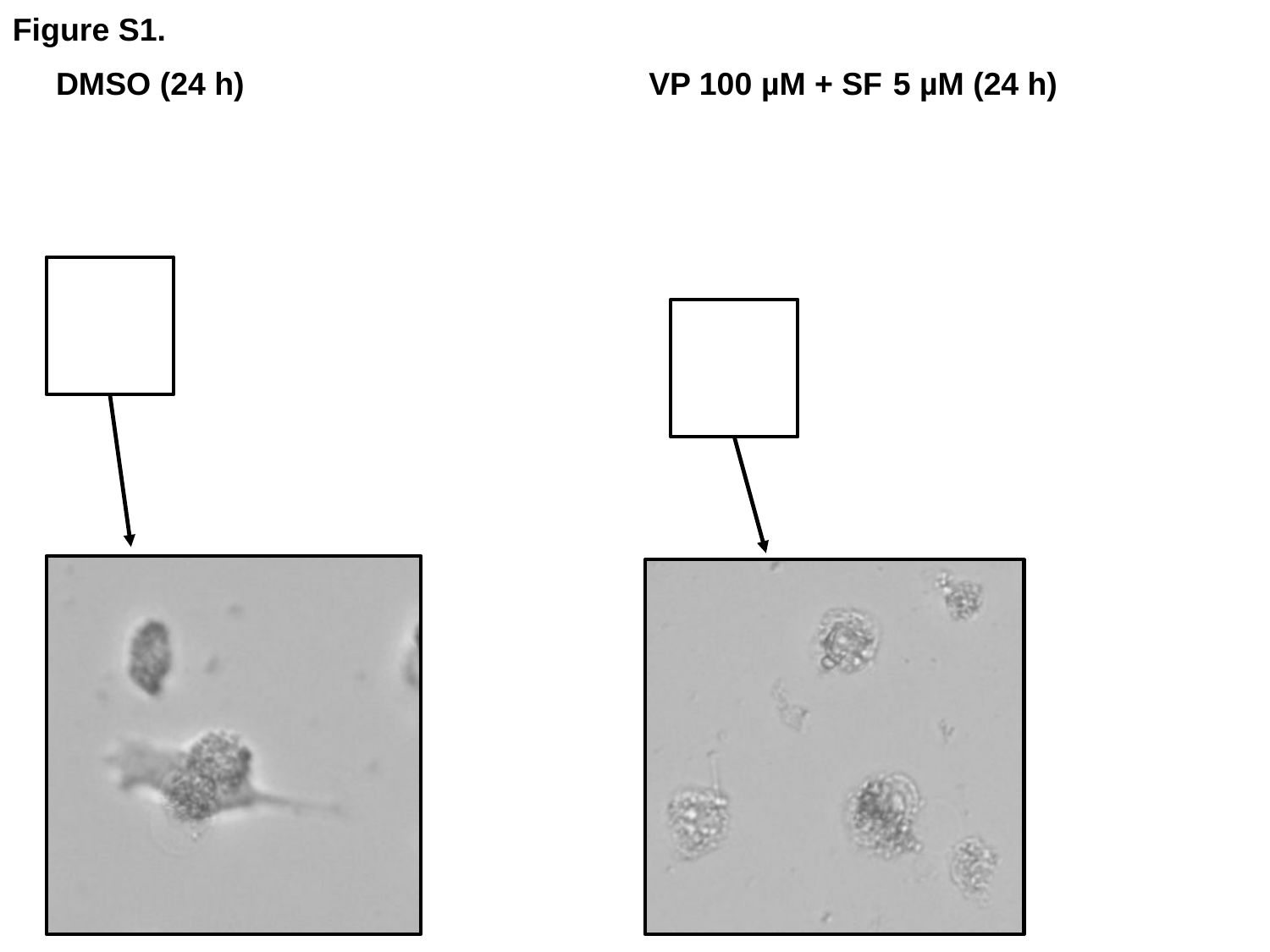

Figure S1.

Supplement: Supplementary file 2 [file 5987015.f2.pptx]

## Slide 1
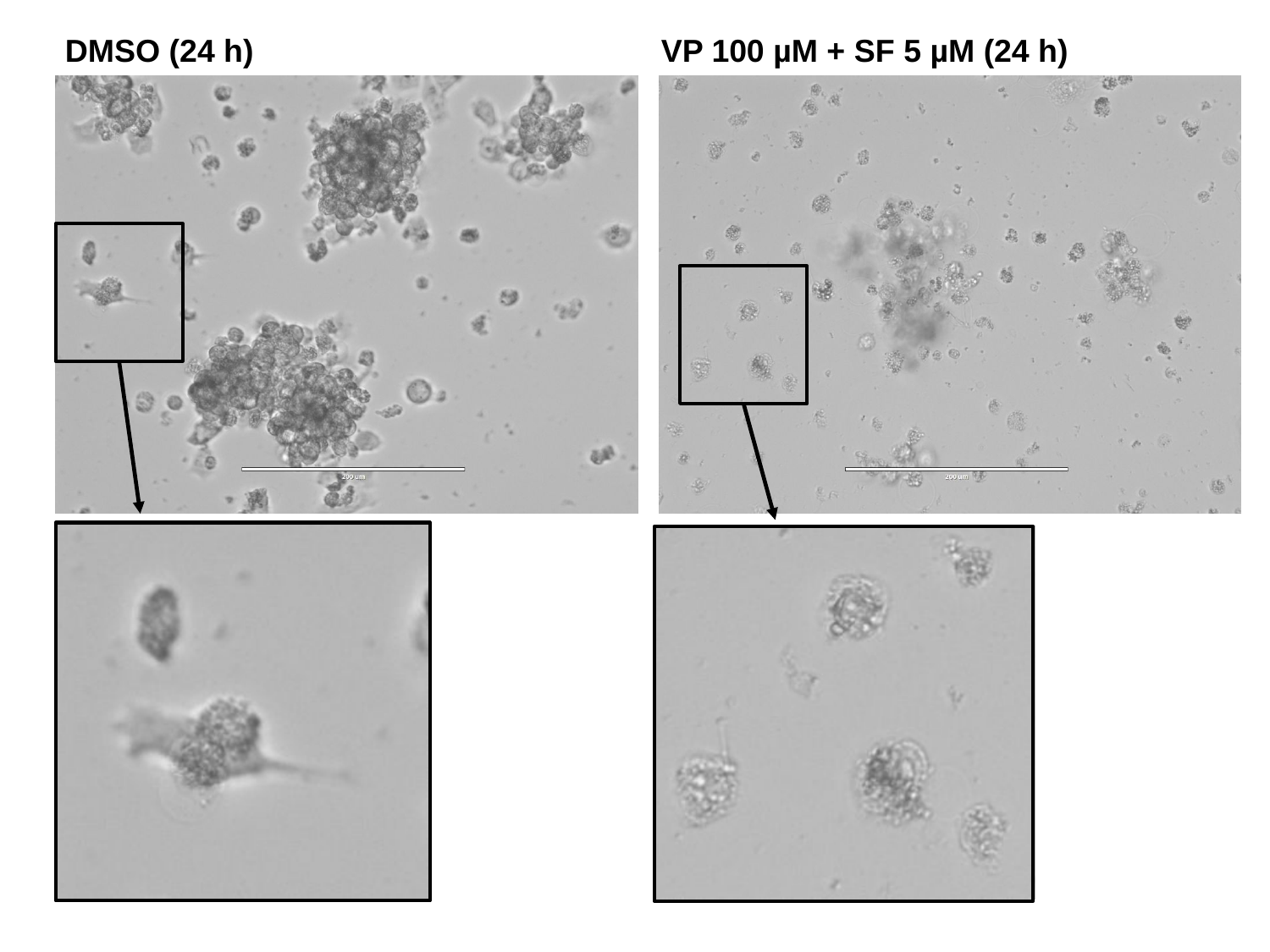

DMSO (24 h) VP 100 µM + SF 5 µM (24 h)

Supplement: Supplementary file 3 [file 5987015.f3.pptx]

## Slide 1
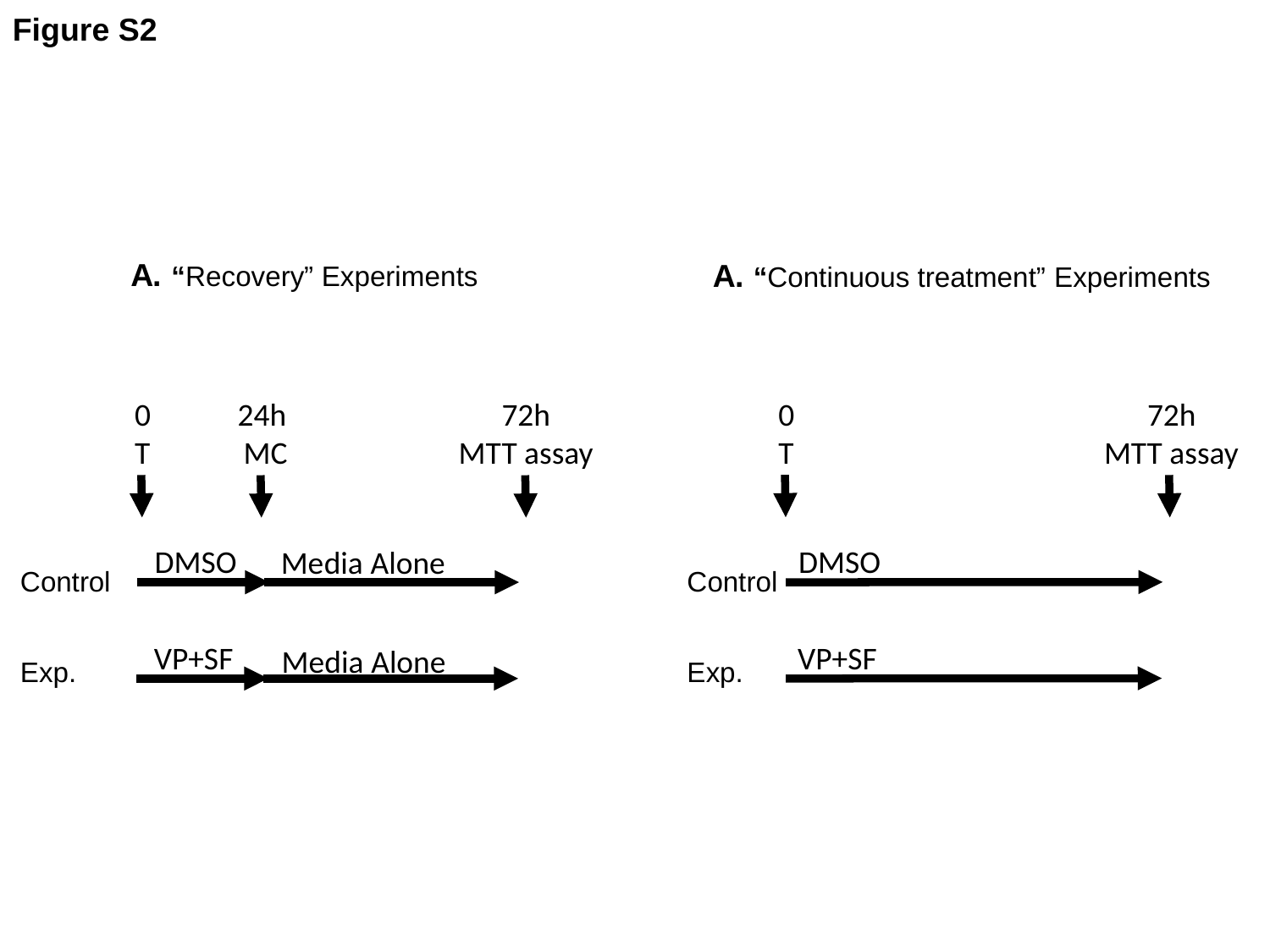

Figure S2

Supplement: Supplementary file 4 [file 5987015.f4.pptx]

## Slide 1
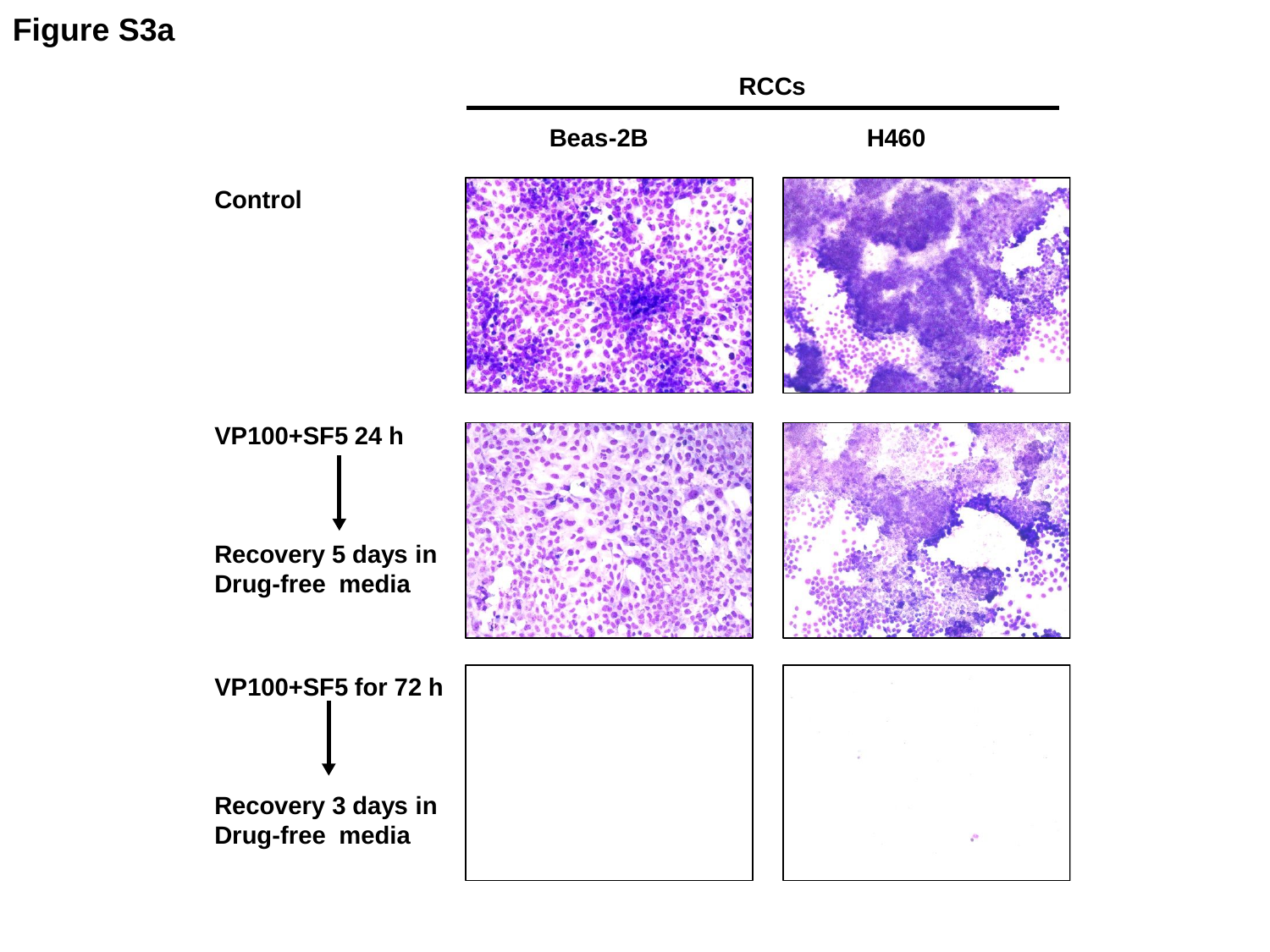

Figure S3a

Supplement: Supplementary file 6 [file 5987015.f6.pptx]

## Slide 1
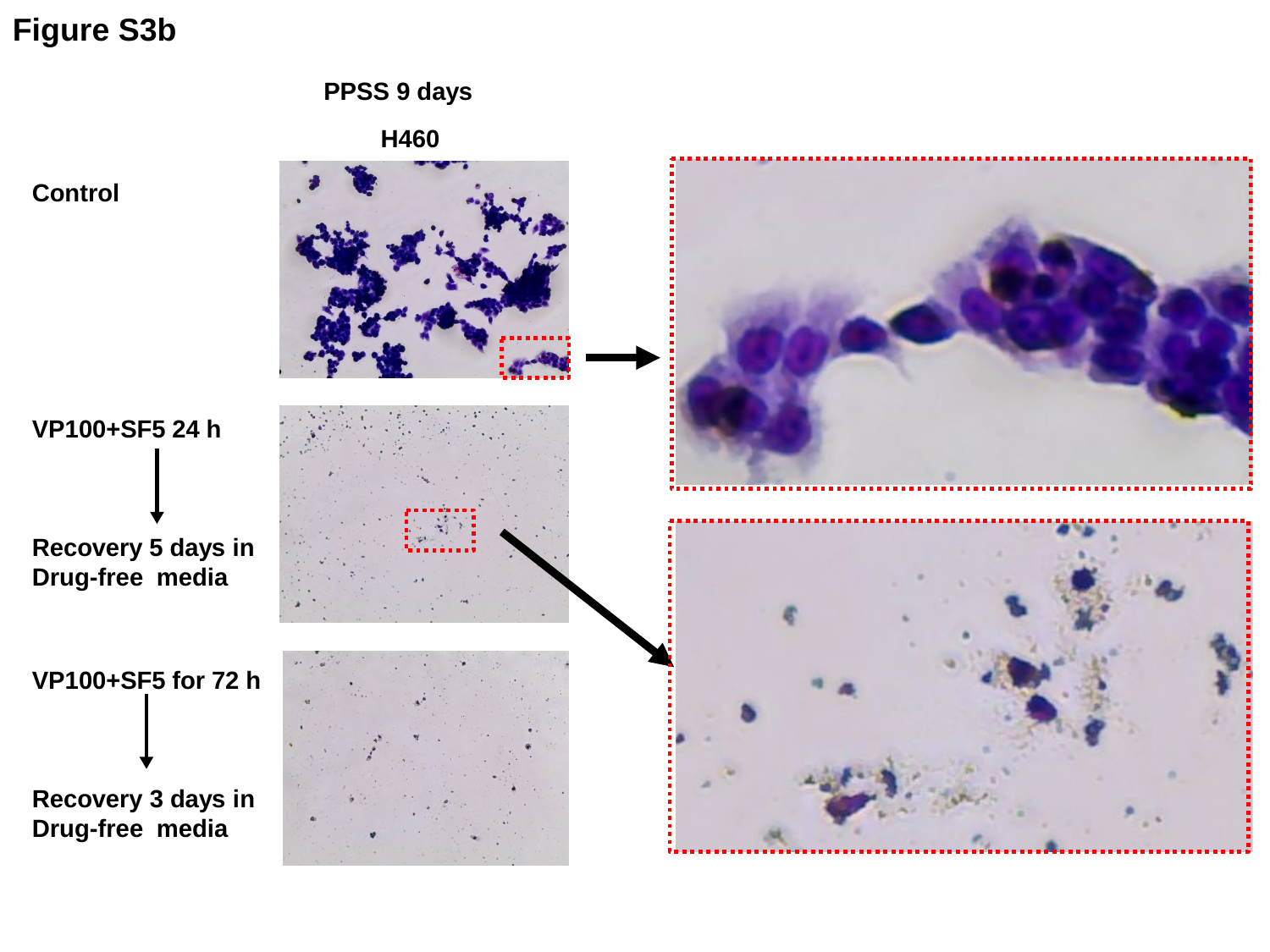

Figure S3b

Supplement: Supplementary file 8 [file 5987015.f8.pptx]

## Slide 1
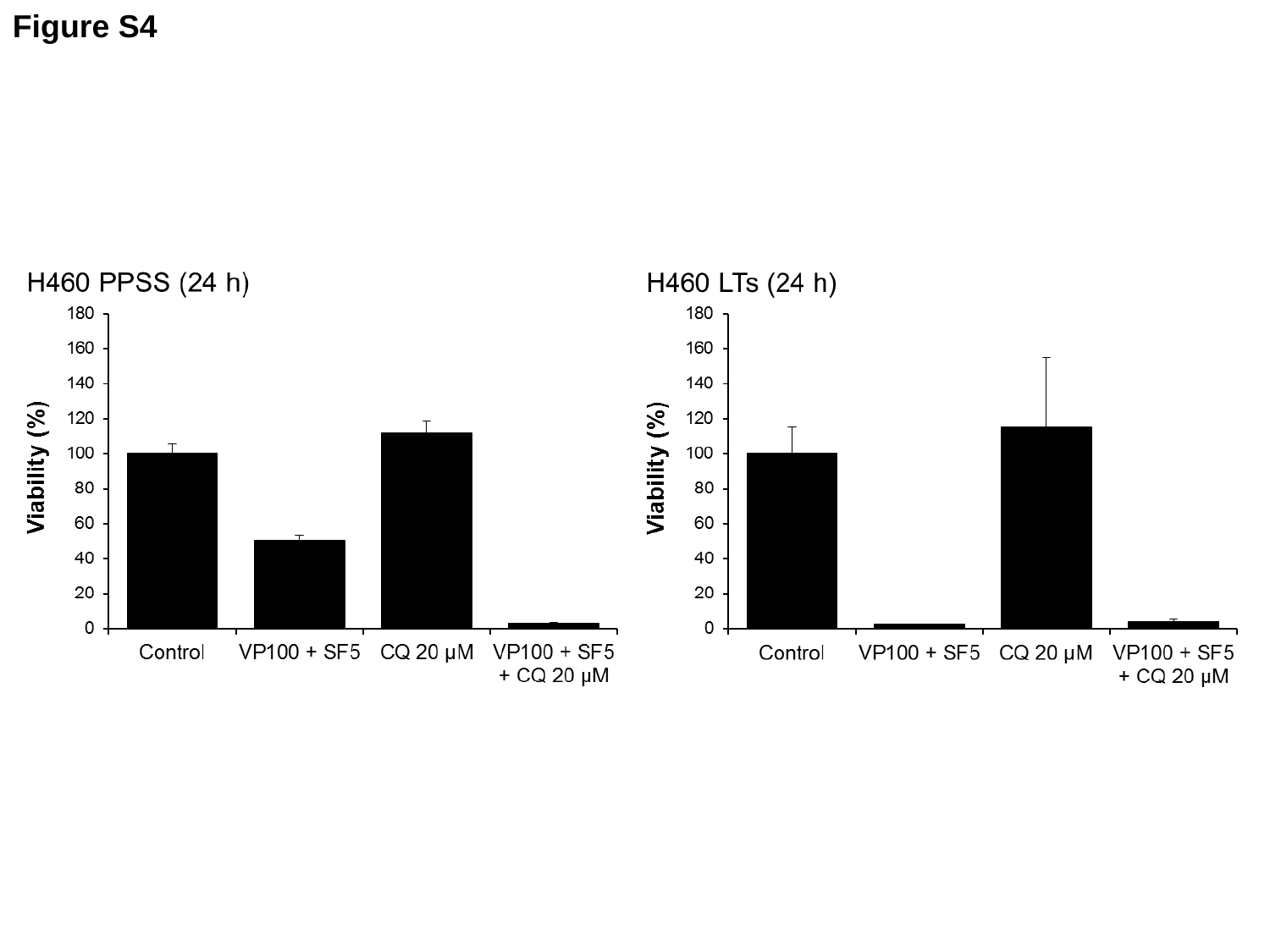

Figure S4
VP (µM) - 100 - - - 100 100 100
SF (µM) - 5 - 5 - 5 5 5
zVAD (µM) ) - - - 10 10 - 10 10
Nec1 (µM) ) - - 50 - 50 50 - 50
VP (µM) - 100 - - - 100 100 100
SF (µM) - 5 - 5 - 5 5 5
zVAD (µM) ) - - - 10 10 - 10 10
Nec1 (µM) ) - - 50 - 50 50 - 50

Supplement: Supplementary file 10 [file 5987015.f10.pptx]

## Slide 1
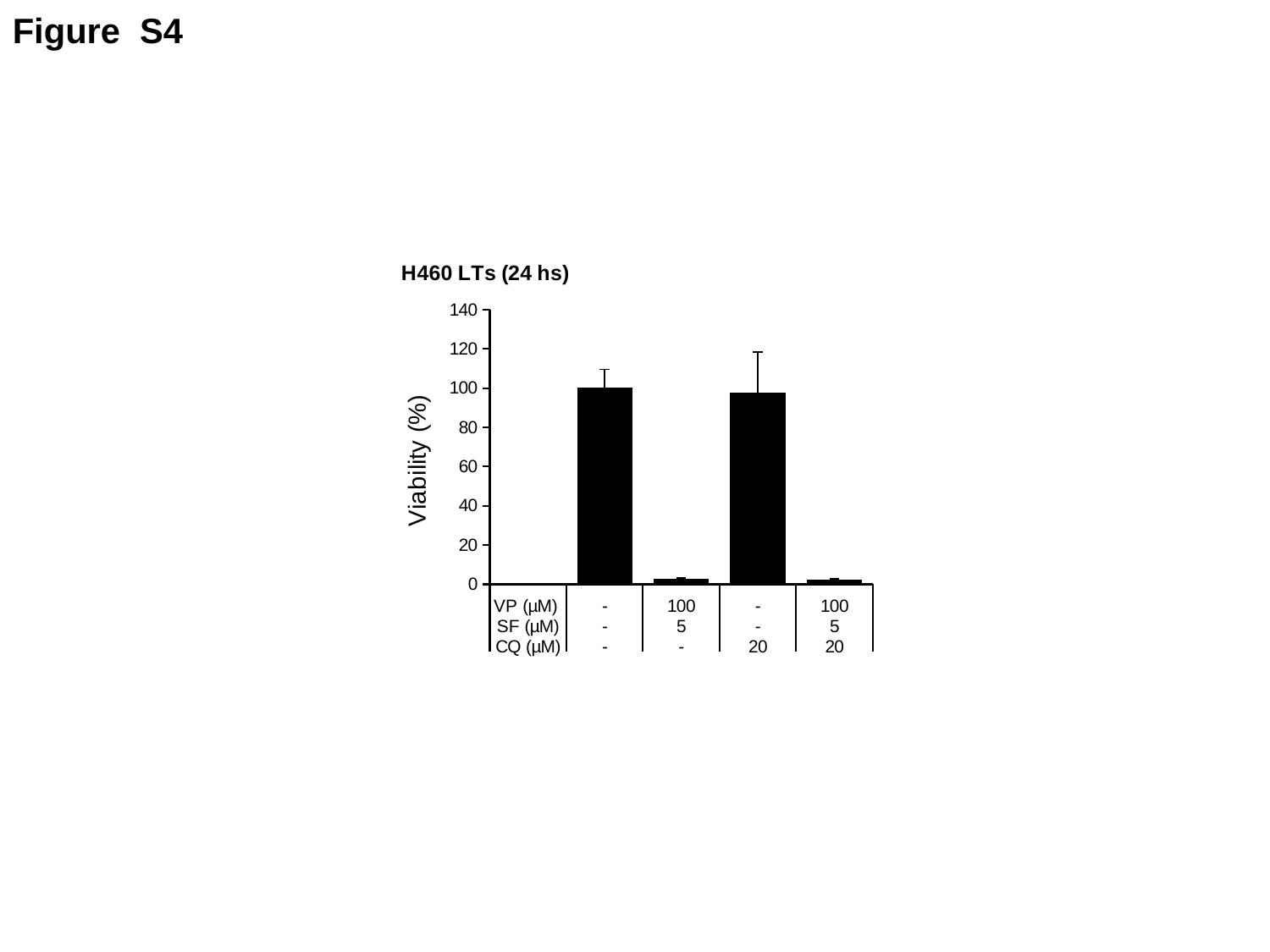

Figure S4
### Chart: H460 LTs (24 hs)
| Category | |
|---|---|
| VP (µM) | 0.0 |
| - | 100.0 |
| 100 | 2.6594719599152046 |
| - | 97.28271343226055 |
| 100 | 2.2933127770283277 |

Supplement: Supplementary file 11 [file 5987015.f11.pptx]
