# Supplementary material for: Selective and Irreversible Induction of Necroptotic Cell Death in Lung Tumorspheres by Short-Term Exposure to Verapamil in Combination with Sorafenib
Source: Stem Cells Int. 2017 Oct 19;2017:5987015. doi: 10.1155/2017/5987015 (PMC5671752; doi:10.1155/2017/5987015)
Supplement: Supplementary file 5 [file 5987015.f5.pptx]

## Slide 1
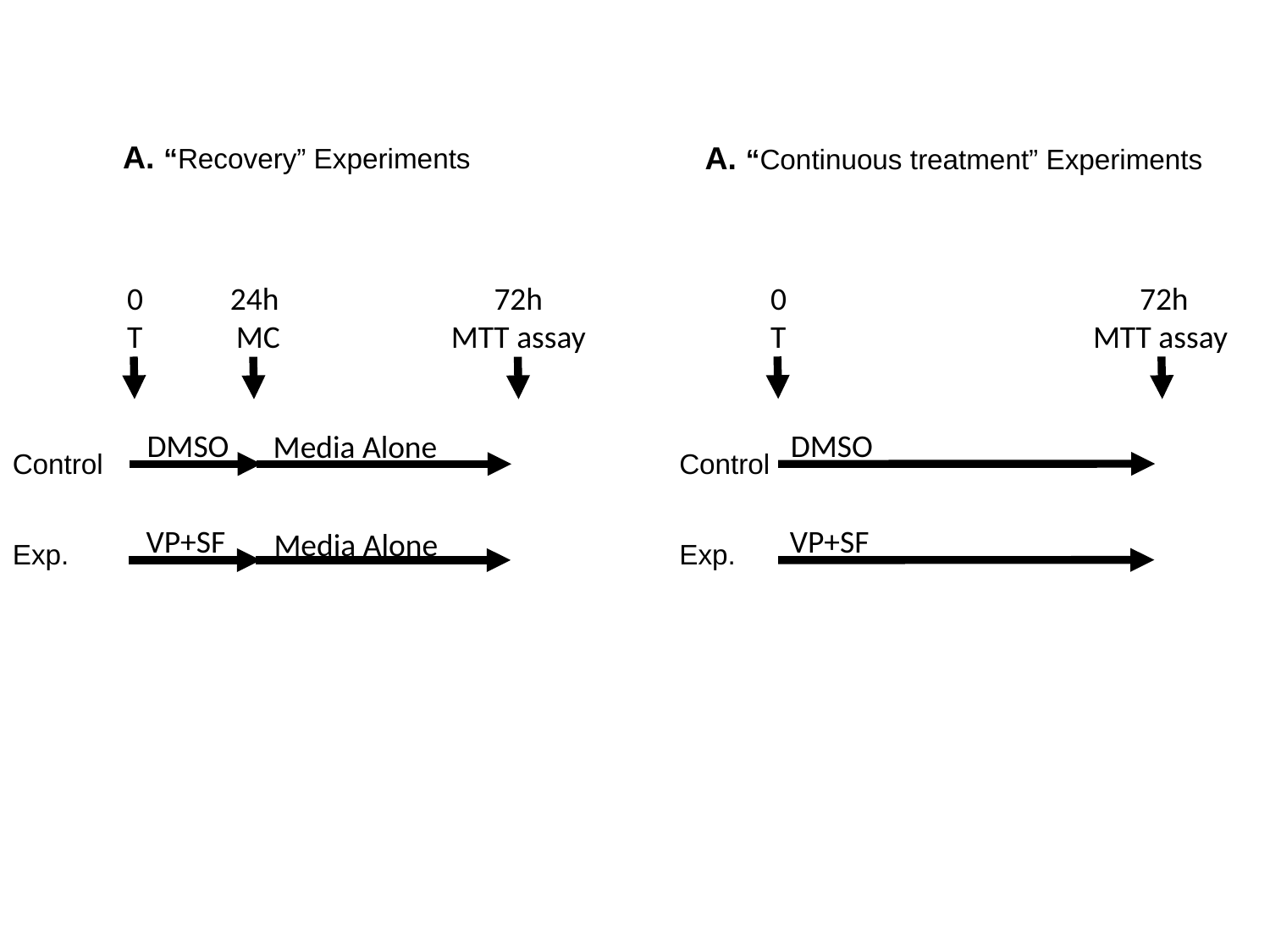

A. “Recovery” Experiments
A. “Continuous treatment” Experiments
0
T
72h
MTT assay
0
T
24h
 MC
72h
MTT assay
DMSO
DMSO
Media Alone
Control
Control
VP+SF
VP+SF
Media Alone
Exp.
Exp.
