# Supplementary material for: Selective and Irreversible Induction of Necroptotic Cell Death in Lung Tumorspheres by Short-Term Exposure to Verapamil in Combination with Sorafenib
Source: Stem Cells Int. 2017 Oct 19;2017:5987015. doi: 10.1155/2017/5987015 (PMC5671752; doi:10.1155/2017/5987015)
Supplement: Supplementary file 7 [file 5987015.f7.pptx]

## Slide 1
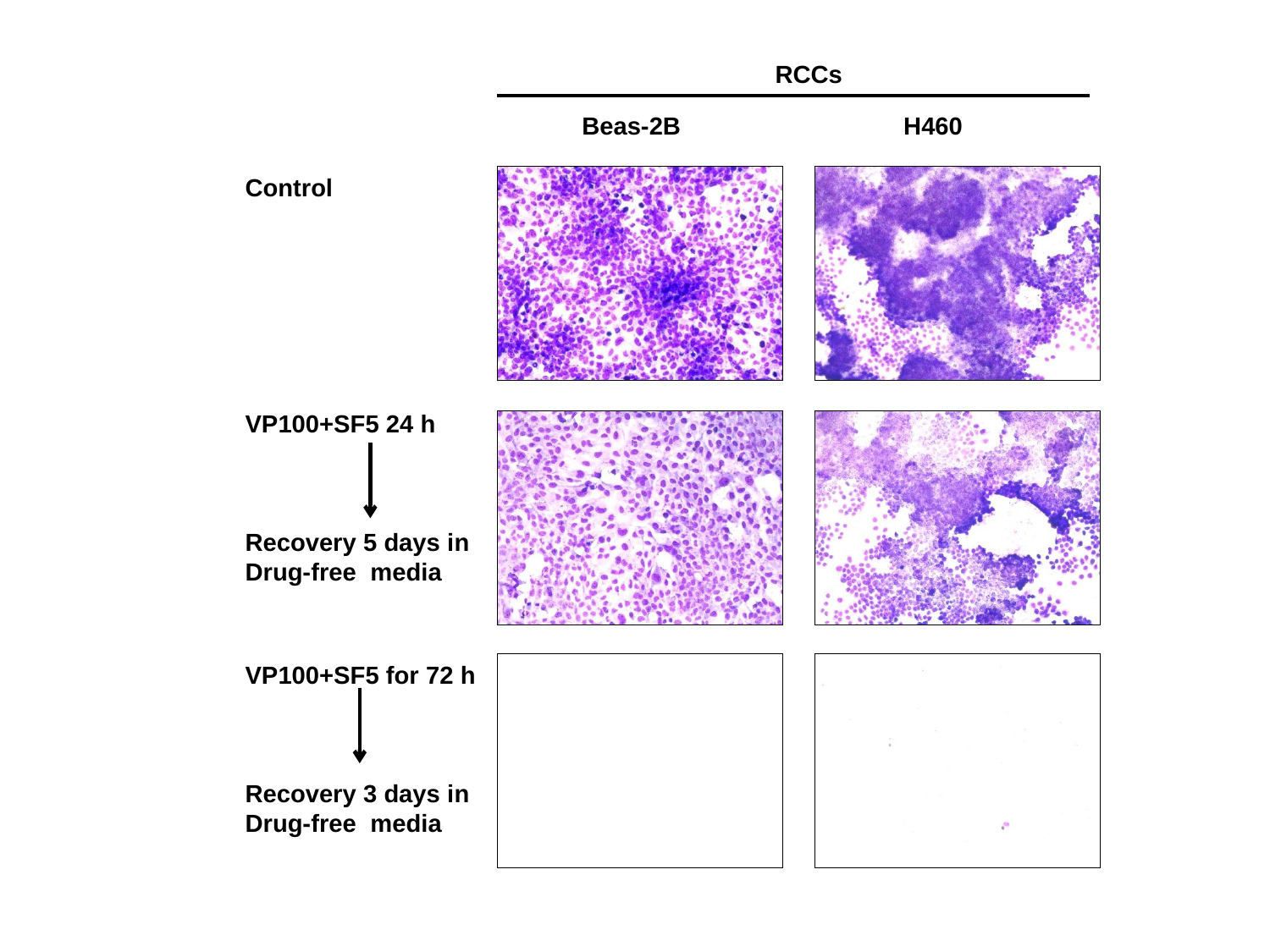

RCCs
 Beas-2B H460
Control
VP100+SF5 24 h
Recovery 5 days in Drug-free media
VP100+SF5 for 72 h
Recovery 3 days in Drug-free media
